# Development of an online UV–visible microspectrophotometer for a macromolecular crystallography beamline

**DOI:** 10.1107/S0909049513022887

**Published:** 2013-10-02

**Authors:** Nobutaka Shimizu, Tetsuya Shimizu, Seiki Baba, Kazuya Hasegawa, Masaki Yamamoto, Takashi Kumasaka

**Affiliations:** a Structural Biology Group, SPring-8/JASRI, 1-1-1 Koto, Sayo-cho, Sayo-gun, Hyogo 679-5198, Japan; b Institute of Materials Structure Science, High Energy Accelerator Research Organization (KEK), 1-1 Oho, Tsukuba, Ibaraki 305-0801, Japan; c RIKEN SPring-8 Center, 1-1-1 Koto, Sayo-cho, Sayo-gun, Hyogo 679-5148, Japan

**Keywords:** UV–visible spectroscopy, protein crystallography, radiation damage, microspectroscopy, SPring-8

## Abstract

Measurement of the UV–visible absorption spectrum is a convenient technique for detecting chemical changes of proteins, and it is therefore useful to combine spectroscopy and diffraction studies. An online microspectrophotometer for the UV–visible region was developed and installed on the macromolecular crystallography beamline, BL38B1, at SPring-8. This spectrophotometer is equipped with a difference dispersive double monochromator, a mercury–xenon lamp as the light source, and a photomultiplier as the detector. The optical path is mostly constructed using mirrors, in order to obtain high brightness in the UV region, and the confocal optics are assembled using a cross-slit diaphragm like an iris to eliminate stray light. This system can measure optical densities up to a maximum of 4.0. To study the effect of radiation damage, preliminary measurements of glucose isomerase and thaumatin crystals were conducted in the UV region. Spectral changes dependent on X-ray dose were observed at around 280 nm, suggesting that structural changes involving Trp or Tyr residues occurred in the protein crystal. In the case of the thaumatin crystal, a broad peak around 400 nm was also generated after X-ray irradiation, suggesting the cleavage of a disulfide bond. Dose-dependent spectral changes were also observed in cryo-solutions alone, and these changes differed with the composition of the cryo-solution. These responses in the UV region are informative regarding the state of the sample; consequently, this device might be useful for X-ray crystallography.

## Abstract 2

An online UV–visible microspectrophotometer has been developed for the macromolecular crystallography beamline at SPring-8. Details of this spectrophotometer are reported.

## 1. Introduction

Spectroscopy is a widely used and effective technique for estimating the structural states of proteins. Some proteins bind metal atoms and/or small organic molecules that absorb in the visible region, and chemical changes of these specific moieties can be detected by spectroscopy. Such chemical changes often effect structural changes of proteins, even within crystals. Therefore, it is useful to combine spectroscopy and diffraction studies. To measure tiny crystal samples, many kinds of online microspectrophotometer have been developed and introduced into several synchrotron beamlines dedicated to macromolecular crystallography (Sakai *et al.*, 2002 ▶; Weik *et al.*, 2002 ▶; Beitlich *et al.*, 2007 ▶; Owen *et al.*, 2009 ▶).

Even in proteins that do not contain any chromophore groups, some amino acids absorb in the UV region. In particular, unlike other amino acids, Trp and Tyr have significant absorption peaks around 280 nm. However, the absorbance in the UV region of protein crystals is generally too high to be measured by conventional microspectrophotometers, so it is necessary to develop spectrophotometers that can measure large absorbances. Moreover, most of the spectrophotometers mentioned above can measure absorption only at wavelengths greater than 300 nm, *i.e.* in the visible region. These technical challenges are related to the complexity of assembling alternative optical components that can accommodate and process UV light.

Structural changes induced by X-ray irradiation, *i.e.* radiation damage, are the most serious problem in protein crystallography using high-flux X-rays generated by synchrotron radiation. The mechanism of radiation damage remains elusive, even though the associated structural changes can often be observed directly in conclusive electron-density maps (Ravelli & McSweeney, 2000 ▶; Weik *et al.*, 2000 ▶; Matsui *et al.*, 2002 ▶; Shimizu *et al.*, 2007 ▶). In order to observe the spectral changes in the UV region and evaluate the effects of radiation damage on amino acids, we developed and installed a new online UV–visible microspectrophotometer at SPring-8 BL38B1.

## 2. Materials and methods

### 2.1. Microspectrophotometer

**Figure 1:**
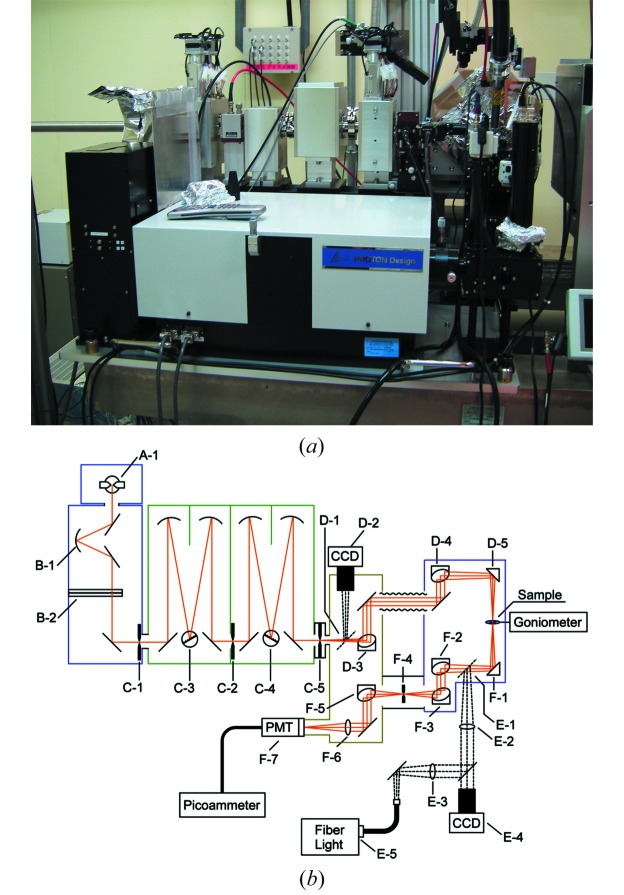
Photograph (*a*) and block diagram sketch (*b*) of the online UV–visible microspectrophotometer. A-1: Hg-Xe lamp; B-1: concave mirrors; B-2: two sets of filter folders; C-1,2: first and second slits in the horizontal direction; C-3,4: first and second diffraction gratings; C-5: third slit in the horizontal and vertical direction; D-1: flat mirror for changing the optical path (insertion and removal); D-2: CCD camera for tuning the monochromator; D-3,4: aspherical mirror; D-5: prism flat mirror; E-1: flat mirror for changing optical path (insertion and removal); E-2,3: convex lens; E-4: CCD camera for centering of the sample crystal; E-5: fiber-optic light for illuminating the sample; F-1: prism flat mirror; F-2,3: aspherical mirror; F-4: cross-slit diaphragm for confocal system; F-5: aspherical mirror; F-6: convex lens; F-7: photomultiplier tube. The beam size of the measurement light is defined by slit C-5, and can be adjusted to a minimum of 50 µm × 50 µm.

The microspectrophotometer installed at SPring-8 BL38B1 (Fig. 1*a* ▶) consists of four sections: the lamp housing, the monochromator, the measurement unit and the detector unit. The whole set-up of this microspectrophotometer is shown as a block diagrammatic sketch in Fig. 1(*b*) ▶. This spectrophotometer was equipped with a difference dispersive double monochromator mounted in Czerny–Turner configuration (PDPT0320, PHOTON design). Such double monochromators achieve very low levels of stray light. Two types of diffraction gratings (2400 grooves mm^−1^, 53006BK01-150R, and 1200 grooves mm^−1^, 53006BK01-280R, Newport) were installed in the monochromator in order to optimize to each wavelength region of UV (250–500 nm) and visible (350–700 nm) light. A 200 W mercury–xenon (Hg–Xe) lamp (L2423, Hamamatsu Photonics) and a photomultiplier tube with multialkali photocathode material (R374, Hamamatsu Photonics) were selected as the light source and detector, respectively. The photocurrent from the detector is read by a picoammeter (Model 486, Keithley). The monochromator and the picoammeter are controlled from original software on a remote computer, connected with a GPIB (PCMCIA-GPIB, National Instruments) through the multi-slot bus-bridge (CBI-8521, CBI-U06D, Interface). In order to acquire high brightness in the UV region, essentially the entire optics system was constructed using mirrors. Some slits were placed at the effective focus of aspherical mirrors, and the confocal system was constructed using a cross-slit diaphragm like an iris at the front of the detector, in order to eliminate stray light generated by not only the optical components but also the cryo-solution around the sample crystal. Two CCD cameras were installed to adjust and align these components and the sample crystal. The position of the X-ray beam is identical to the light path of the spectrophotometer. The measurement light of the spectrophotometer is focused on the focal point of the camera for the centering of a sample crystal in the diffraction experiment. Therefore, the crystal can be simultaneously aligned to the optimal position against both the X-ray beam and the measurement light of the spectrophotometer.

The spectrophotometer was integrated into the diffractometer of BL38B1 (Fig. 1*a* ▶), and it is controlled by the beamline control software, *BSS* (Ueno *et al.*, 2005 ▶). Owing to the housing size of the measurement unit, it must be removed from the sample position during mounting of a crystal and diffraction measurements. Therefore, the scanning of the absorption spectrum cannot be performed simultaneously with an X-ray diffraction measurement; however, the two modes, *i.e.* elevation and removal of the measurement unit, can be quickly switched by *BSS*. Even during mode switching, sample crystals on the goniometer are kept cooled by an N_2_ gas stream at 100 K. When carrying out spectral measurements, the experimental environment is made as dark as possible by dimming all the illumination lamps and hanging a blackout curtain. Light leaked from the photosensors of the automatic stage and lamp housing is also shaded sufficiently that absorbance can be measured with high dynamic range.

### 2.2. Crystallization for microspectroscopy

**Figure 2:**
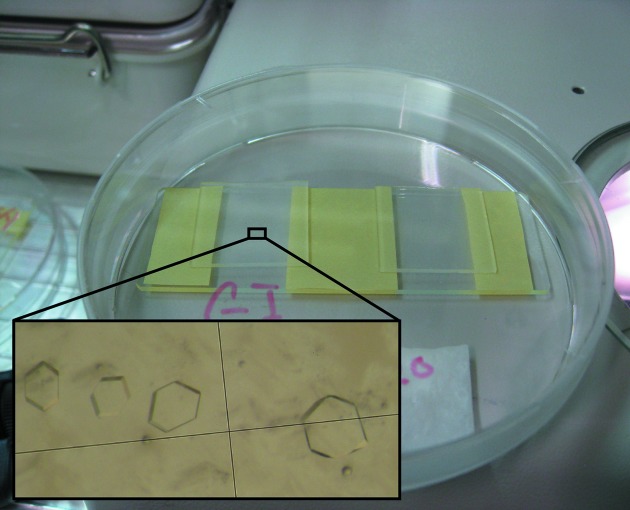
Photograph of the crystallization of glucose isomerase for microspectroscopy. In order to prevent the sample solution from drying, crystallization was performed within a sealed petri dish containing cotton wool saturated with precipitant. The inset displays thin board-shaped crystals with dimensions of around 0.3–0.5 mm.

Glucose isomerase and thaumatin crystals were used in this study. Glucose isomerase (HR7-100, Hampton Research) was crystallized at 293 K and pH 7.0 with 15% (*w*/*v*) 2-methyl-2,4-pentanediol (MPD) and 0.2 *M* magnesium chloride after being prepared for crystallization according to the user guide for glucose isomerase supplied by Hampton Research. Thaumatin powder (T7638, Sigma-Aldrich) was dissolved in 20 m*M* HEPES to a concentration of ∼35 mg ml^−1^, and crystallized at 293 K and pH 7.3 with 0.9 *M* sodium tartrate, 100 m*M* HEPES and 15% glycerol. In order to optimize the absorbance of the crystals in the UV region, we used a crystallization method to limit the size of the crystals. A protein crystal has high absorbance in the UV region, because the orientations of all molecules in the crystal are uniform as a result of crystallization. Because absorbance increases in proportion to the thickness of the crystal, sample crystals were grown in droplets sandwiched between two siliconized glass plates in order to limit their thickness as described by Kort *et al.* (2003 ▶). Fig. 2 ▶ shows the crystallization set-up and the resultant crystals of glucose isomerase. Thin board-shape crystals with dimensions of 0.3–0.5 mm were obtained using a polyester tape with a thickness of 0.02 mm (tape No. 74, 3M Company) as a spacer; this approach succeeded in suppressing the absorbance of the crystal at 280 nm to an optical density (OD) of ∼1.9.

### 2.3. Measurement of UV spectral change by X-ray irradiation

X-ray irradiation experiments were performed at BL38B1 (Kawano *et al.*, 2010 ▶). The X-ray wavelength was set to 1 Å, and the beam size at the sample position was 0.18 mm × 0.23 mm. The X-ray intensity was chosen based on the absorbed dose calculated by the program *RADDOSE* (Murray *et al.*, 2004 ▶; Paithankar *et al.*, 2009 ▶; Paithankar & Garman, 2010 ▶). When a crystal of glucose isomerase with dimensions 0.43 mm × 0.43 mm × 0.02 mm was irradiated with X-rays of 1 Å at BL38B1, the absorbed dose was estimated as 3.7 × 10^−3^ MGy s^−1^. The X-ray intensity was gradually raised by changing the thickness of the aluminium attenuator during repeated 1 s irradiations. In the first measurement depicted in Fig. 4(*a*), the crystal did not oscillate during the X-ray irradiation, in order to fix the incidence angle of the X-rays with respect to the crystal, and the largest area of the crystal always faced the X-rays. For two other crystals of almost the same size and the samples of cryo-solutions, oscillation measurements were repeatedly performed; over the course of 180 images collected 1° apart, the total doses absorbed by the crystal were 2 MGy for glucose isomerase and 2.7 MGy for thaumatin. Figs. 4(*b*)–4(*d*) demonstrate the absorption spectra before and after X-ray irradiation at each total dose.

In order to compare the absorbance and perform calculations based on the absorbance, it is essential to keep the thickness of a sample constant. Litholoops (round type, Molecular Dimensions), 0.5 mm in diameter, were chosen as sample loops. The thickness of the litholoops, 0.02 mm, is identical to that of the crystal. When measuring the absorption spectra of the crystals and the cryo-solutions, the largest area of the sample faced to the measurement light orthogonally. The sample thickness is always controlled to be the same as the thickness of the loop when mounted on the goniometer. It can be confirmed using the CCD camera for the sample centering. Therefore, the sample thickness was maintained at 0.02 mm in all samples.

## 3. Results and discussion

### 3.1. Properties of the microspectrophotometer

**Figure 3:**
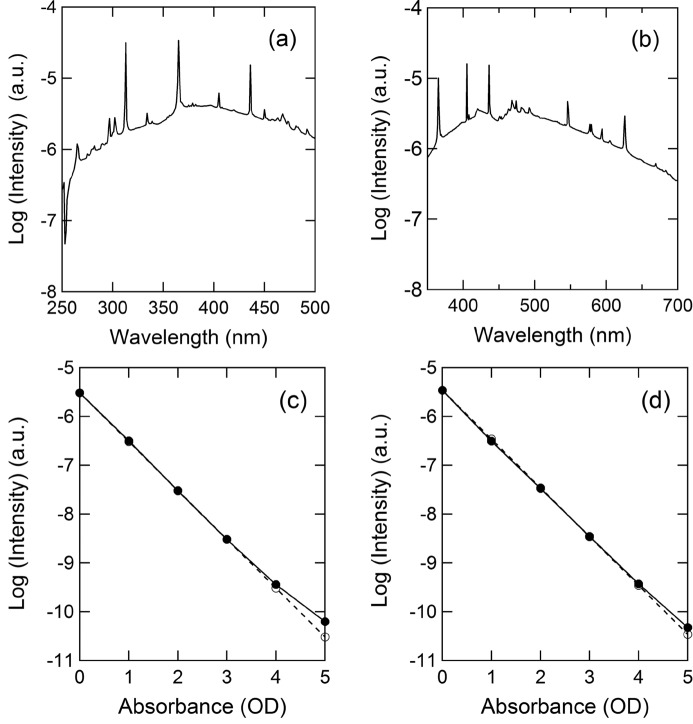
Scanning blank spectrum of the microspectrophotometer measured in the UV region (*a*) and visible region (*b*). The optimal diffraction grating for each wavelength region is installed in this monochromator. (*c*) and (*d*) Linearity between the detector signal and the intensity of incident light. The measurement wavelengths were 300 nm (*c*) and 440 nm (*d*). Closed circles show the observed values, and open circles show the theoretical values, which were obtained after subtracting the dark current.

The baseline spectrum for a blank sample is reasonable for measuring protein crystals in each wavelength region [Figs. 3(*a*) and 3(*b*) ▶]. Although some sharp peaks representing the emission lines of the Hg–Xe lamp were observed, the monochromator can be tuned based on the wavelength of these lines. The photocurrent was adjusted so that the value at 436 nm was ∼1.5 × 10^−5^ A (15 µA). The detector was operated at an applied voltage of −1000 V.

The linearity between the detector signal and the intensity of incident light was maintained up to an OD of 4.0, as shown in Figs. 3(*c*) and 3(*d*) ▶. In the graphs, the logarithm of the photocurrent is plotted against absorbance. The measurement light was attenuated by UV–visible neutral density filters (47511-L, Edmund). The measurement wavelengths were 300 nm in the UV region and 440 nm in the visible region. Although the linearity in the UV region was slightly lower than that in the visible region, the results suggest that the absorption spectrum can be measured up to ∼4.0 OD in the range 250–700 nm.

The spectrophotometer system works well and can be applied to structural and spectroscopic analyses of proteins. For instance, dose-dependent reduction of siroheme in tobacco nitrite reductase was observed using this device (Nakano *et al.*, 2012 ▶). After irradiation with doses of 1.6 MGy or more, a positional shift of a water molecule close to the siroheme and a spectrum shift of the α-band peak of siroheme (568 nm to 552 nm) could be detected. The fact that the reduction of the siroheme induced the positional shift suggested some details of the mechanism of the catalytic reaction.

### 3.2. Preliminary measurement for radiation damage

**Figure 4:**
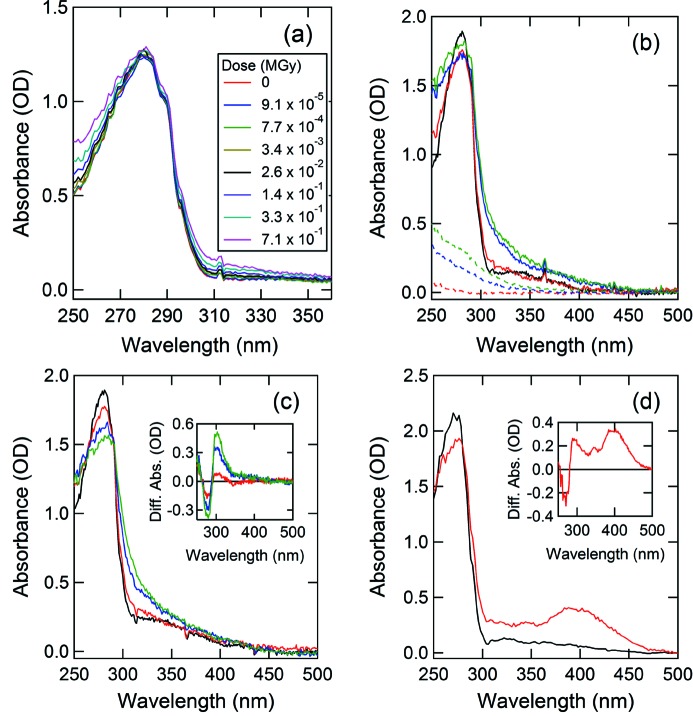
Absorption spectra of sample crystals measured before and after X-ray irradiation. (*a*), (*b*), (*c*) are results for glucose isomerase, and (*d*) is a result for thaumatin. (*a*) Absorption spectra measured under the condition at low doses. The dose was increased stepwise. (*b*) High-dose measurements were performed on the crystal (solid line) and the cryo-solution (broken line). Black, red, blue and green lines indicate absorbed doses of 0, 2, 20 and 40 MGy, respectively. (*c*) The net absorption spectra calculated by subtracting the absorbance of the cryo-solution from that of the crystal. The colors indicate the same dose conditions as in (*b*). The inset figure shows the difference spectra obtained by subtracting the absorbance at 0 MGy from the absorbances at 2, 20 and 40 MGy. (*d*) Absorption spectrum after subtracting the absorbance of the cryo-solution from that of the thaumatin crystal. Black and red indicate absorbed doses of 0 and 2.7 MGy, respectively. The inset figure shows the difference spectra obtained by subtracting the absorbance at 0 MGy from the absorbance at 2.7 MGy.

A dose-dependent spectral change of a glucose isomerase crystal was observed in the UV region. The first change in the spectrum was observed at 3.4 × 10^−3^ MGy irradiation at around 250 and 320 nm (Fig. 4*a* ▶). This difference was small, but the trend in the spectral change was maintained. Although further changes in absorbance at ∼250 and ∼320 nm were observed, the baseline from 240–400 nm seemed to gradually shift upward as the dose increased.

Solvent effects are important to interpretations of these results. In order to clarify this shift of the baseline, the absorption spectrum of the cryo-solution was also measured after X-ray irradiation under the same conditions (Fig. 4*b* ▶). MPD was selected as the cryoprotectant for glucose isomerase, and its concentration was adjusted to 25% (*w*/*v*). The absorbance of cryo-solution clearly increased in the wavelength range from 240 to 350 nm as the dose increased. This absorbance change was almost identical to the spectral shift observed in the crystal. These observations suggest that, in order to obtain the absorption spectrum of only the protein in the crystal, it is necessary to subtract the absorbance of the cryo-solution from that of the crystal in the cryo-solution. The net spectra after subtraction can be seen in Fig. 4(*c*) ▶.

The net spectra from the glucose isomerase crystal showed that the absorbance at 280 nm decreased, whereas the absorbance at 250–300 nm increased, and the isosbestic point was observed at 290 nm. The absorbance change around 280 nm might indicate the structural change of a Trp or a Tyr, because glucose isomerase has six Trp and nine Tyr residues (among 388 total residues).

On the other hand, the absorption spectrum of the thaumatin crystal was different from that of glucose isomerase. For thaumatin, 30% (*w*/*v*) glycerol was used as the cryoprotectant solution. The absorbance at 280 nm also decreased, as in the case of glucose isomerase, and a broad peak around 400 nm was generated after X-ray irradiation (2.7 MGy), suggesting that a disulfide bond of thaumatin was damaged and broken (Weik *et al.*, 2002 ▶; Southworth-Davies & Garman, 2007 ▶). In the case of thaumatin, difference spectra at 20 MGy or more could not be calculated because the spectral change of the cryo-solution became too large at high doses. These results suggest that the effects of radiation damage change with the composition of the cryo-solution (McGeehan *et al.*, 2009 ▶).

## 4. Conclusion

We have developed and installed an online microspectro­photometer at beamline BL38B1 of SPring-8. This device can measure the absorption spectrum in the UV region, and is open to users. High dynamic range is achieved in the UV wavelength region by a combination of mirror and confocal optics. However, because the absorbance of protein in the UV region is quite large, it is important to control crystal thickness. Absorption-spectrum changes due to radiation damage to protein crystals were successfully observed at around 280 nm. These changes might involve structural changes of Trp or Tyr residues; however, crystallographic analysis was not performed in this study. Comparisons between absorption spectra and electron-density maps should be helpful in elucidating the structural changes in proteins that arise as a result of radiation damage.
